# The problem with picking: Permittance, escape and shame in problematic skin picking

**DOI:** 10.1111/papt.12427

**Published:** 2022-09-19

**Authors:** Suzy Anderson, Victoria Clarke, Zoe Thomas

**Affiliations:** ^1^ School of Social Sciences, College of Health, Science and Society University of the West of England Bristol UK

**Keywords:** appearance, attention, cognition, distress, emotion regulation, mental health, qualitative, therapy

## Abstract

**Objectives:**

Problematic skin picking (SP) is a poorly understood experience characterised by a drive to pick the skin and related psychosocial impact. In the DSM‐5, problematic SP is classified as ‘excoriation (skin picking) disorder’. The aim of this article is to present a rare qualitative perspective on the lived experience of problematic SP, prioritising participants' voices and sense‐making.

**Design:**

An in‐depth qualitative study of individuals who self‐identified as picking their skin problematically and experienced related distress.

**Methods:**

Seventeen UK‐based participants were recruited online and interviewed about their SP. Participants were given choice of interview modality, including instant messenger platforms, telephone, email and *Skype*, to maximise comfort and improve the accessibility of the study. Transcripts were analysed using thematic analysis.

**Results:**

Three themes offering novel insight into the phenomenology of participants' SP are highlighted and explored: (1) how cognitions and circumstances drove and permitted SP, (2) how participants ‘zoned out’ while SP and the escape or relief that this attentional experience offered and (3) participants' feelings of shame and distress in how they felt their SP may appear to others.

**Conclusions:**

This study contributes in‐depth and novel ideas to the understanding of SP phenomenology and identifies how environmental factors, cognitions, contextual distress and shame may be considerations in therapeutic intervention. It presents the complexity of SP sense‐making and demonstrates the need for individual formulation.

**Practitioner points**

- Problematic skin picking (SP) is a heterogeneous and complex experience. Interventions must be tailored to each individual's needs and experience.
- The frequency and intensity of problematic SP are impacted by how easily picking can be accommodated within daily life. Accordingly, practitioners may work with clients to identify and manage environmental, systemic and cognitive factors that facilitate and contextualise picking behaviours.
- The use of SP in emotional regulation may be explained by the escape that it offers from emotional discomfort. Practitioners should consider clients' emotional experience and support the development of alternative coping strategies.
- Shame defines the experience of SP for many. This shame may be a useful target for therapy, and practitioners need to mindful of taking a non‐judgemental and accepting stance.
- It may be helpful for practitioners to have a pre‐existing understanding of problematic SP to guide exploration.

## BACKGROUND

Picking at skin is a common behaviour, typically giving little cause for concern, that may relate to social bonding, tension reduction, and skin hygiene and appearance (Bohne et al., 2002; Dunbar, 2010; Keuthen et al., 2000; Troisi, 2002). For some, skin picking (SP) may be damaging and problematic, and may be accompanied by distress and dysfunction (e.g. Arnold et al., 1998). The conceptualisation of this more problematic SP has been subject to increased research attention over the past two decades. Research has been almost exclusively quantitative with little representation of subjective, lived experience.

As picking behaviour is not intrinsically troublesome, problematic SP tends to be differentiated from benign SP using criteria more defining of ‘problematic’ than of ‘picking’. In 2013, problematic SP was classified as ‘excoriation (skin picking) disorder’ in the Diagnostic and Statistical Manual of Mental Disorders (DSM‐5) (American Psychiatric Association, 2013; see also Lochner et al., 2012), specifying recurrent SP resulting in lesions, repeated attempts to stop or reduce SP, and that SP causes ‘clinically significant’ distress or functional impairment (APA, 2013). The International Classification of Diseases and Related Health Problems (ICD)‐11 criteria broadly echo these points (World Health Organization, 2019).

SP behaviour is conceptualised and categorised in various ways, including as a ‘displacement behaviour’ (alongside face touching and scratching; Mohiyeddini & Semple, 2013) and as a ‘motor stereotypy’ (in autism and intellectual disability research; Singer, 2009; Sukhodolsky et al., 2008). Within the study of SP as a psychopathology, problematic SP has been grouped with other presentations deemed similar. This includes groupings alongside behaviours such as hair pulling and nail biting as a ‘body focussed repetitive behaviour’ (BFRB; e.g. Teng et al., 2002; within ICD‐11, WHO, 2019), a categorisation describing problematic repetitive behaviours removing small parts of the body (Snorrason et al., 2012). Researchers considering the relationships between SP and hair pulling have found similarities, such as in demographic variables, comorbidities and personality variables (Lochner et al., 2002). Meanwhile, differences have been found in features such as time spent picking/pulling, behavioural triggers, and frequencies of dissociation (Lochner et al., 2002; Odlaug & Grant, 2008a), suggesting that the BFRB grouping does not always denote similarity of experience. Within both DSM‐5 and ICD‐11, problematic SP is categorised as relating to obsessive–compulsive disorder (APA, 2013; WHO, 2019) based on shared features of obsession, compulsion and preoccupation (Abramovitch et al., 2015). Within ICD‐11, problematic SP is then further sub‐categorised as a body‐focused repetitive behaviour (WHO, 2019). Problematic SP has also been considered an issue of impulse control or behavioural addiction (APA, 2000; Odlaug & Grant, 2010), including within earlier editions of the DSM (see DSM‐IV, APA, 2000) and the ICD (see ICD‐10, WHO, 2016). The current discussion of problematic SP will sometimes draw on the groupings discussed above, such as by considering the experience of hair pulling, or the broader category of BFRBs, where information specific to problematic SP is limited.

Varied perspectives on categorisation may reflect diversity in problematic SP presentations. Problematic SP is widely acknowledged to be a heterogeneous in terms of nature, drive and function (e.g. Arnold et al., 2001; Odlaug & Grant, 2010; Siev et al., 2012; Walther et al., 2009); these varied characteristics are briefly discussed below.

### The experience of problematic skin picking

Research shows that problematic SP occurs across multiple bodily sites, with the face, hands, arms, scalp and legs commonly targeted (e.g. Prochwicz et al., 2016; Tucker et al., 2011). SP may occur within daily grooming routines (e.g. Bohne et al., 2002; Deckersbach et al., 2003) or throughout the day (Arnold et al., 1998). Problematic SP may be triggered by perceptual and/or tactile dermatological stimuli (e.g. Neziroglu et al., 2008), though it is not well predicted by the condition of skin alone (Gupta et al., 1996; Prochwicz et al., 2016), and many pick at healthy skin (e.g. Tucker et al., 2011). Problematic SP is often intended to improve the skin's texture or appearance (e.g. Arnold et al., 1998; Keuthen et al., 2000), though these instances are often considered secondary to body dysmorphia (e.g. Grant et al., 2015; McDonald et al., 2020).

Researchers have identified variation in attention and awareness while SP (e.g. Arnold et al., 1998). Walther et al. (2009) suggested ‘focussed’ (SP with awareness), ‘automatic’ (SP without awareness) and ‘mixed’ subtypes of problematic SP to describe these differences. In Anderson and Clarke's (2019) qualitative research, automatic SP seems evident in ‘wandering’ (p. 1777) hands scanning the skin outside of executive control and awareness. Contrastingly, in Deckersbach et al.'s (2003) case report of more focussed SP, the individual described knowingly ‘zooming in on these spots’ (p. 255) using a mirror. SP may begin outside of awareness but become conscious over time (Odlaug & Grant, 2007), and awareness may fade as SP induces a trance‐like state (Wilhelm et al., 1999). Capriotti et al.’s (2015) case series included descriptions of drifting ‘in and out of awareness’ (p. 237), and a participant of Deckersbach et al.'s (2002) case reports described feeling ‘mesmerised’. The participant considered this state to play a part in their regulation of feelings such as sadness, loneliness and anxiety (Deckersbach et al., 2002).

Stress appears to increase body‐focussed movements including scratching and self‐grooming (Troisi, 2002). These movements are theorised to reduce uncertainty (Perrykkad & Hohwy, 2020) and regulate stress (Mohiyeddini & Semple, 2013). Similarly, episodes of problematic SP are reported to be preceded by emotional discomfort, such as stress, tension and feelings of emptiness (e.g. Arnold et al., 1998; Keuthen et al., 2010; Neziroglu et al., 2008). Elevated stress relates to more time spent engaging in BFRBs, higher psychosocial impact and a worse quality of life (Grant et al., 2015), and problematic SP has been associated with trauma (Machado et al., 2018; Özten et al., 2015). SP may be followed by relief and satisfaction (Snorrason et al., 2010; Tucker et al., 2011) and reduced tension (Bohne et al., 2002), a dynamic that has led to theories that problematic SP may regulate or alleviate unpleasant emotions (Roberts et al., 2013; Wilhelm et al., 1999). A participant in Deckersbach et al.'s (2002) case reports described SP to relax and not knowing ‘how to relieve stress in any other way’ (p. 272).

Body focussed repetitive behaviours have been suggested to sometimes relate to low arousal (Penzel, 2003). Internal states such as boredom and impatience appear to trigger BFRBs (Roberts et al., 2015), ‘daydreaming’ is associated with SP (Prochwicz et al., 2016) and some mild skin pickers described SP ‘to give themselves something to do’ (Keuthen et al., 2000, p. 213). Bohne et al. (2002) found SP often occurred when alone at home, though it is unclear whether this might relate to environmental factors or a preference for privacy.

### The impact of problematic skin picking

By clinical definition, SP has problematic consequences spanning physical, psychological and social domains (APA, 2013). Physical damage may include scarring, bleeding and infection (Tucker et al., 2011; Wilhelm et al., 1999), sometimes requiring antibiotic treatment (Odlaug & Grant, 2008b) or surgery (Arnold et al., 1998). Pleasure from SP appears fleeting and is followed by increased reports of shame, guilt, hopelessness, depression, anxiety and humiliation (Flessner & Woods, 2006; Keuthen et al., 2000; Simeon et al., 1997; Snorrason et al., 2010). Problematic SP is often characterised by psychosocial withdrawal, such as staying home or avoiding events (Anderson & Clarke, 2019; Keuthen et al., 2001; Tucker et al., 2011), and makeup and/or clothing are often used to disguise skin damage (Arnold et al., 1998; Wilhelm et al., 1999).

Treatment seeking for problematic SP is low (Neziroglu et al., 2008; Tucker et al., 2011) and problematic SP is considered under‐recognised by medical and psychological professionals (Tucker et al., 2011). Shame and embarrassment may impede recognition and treatment (Bohne et al., 2005). Gallinat et al. (2019) found help seeking to also be inhibited by feelings that SP was not severe enough, uncertainty about who might help and concerns that professionals would not know about problematic SP.

Considering this impact alongside the affective dynamic of SP, distress appears to be both a consequence and precipitator of SP. Grant et al. (2016) suggest three explanations for the relationship: that SP may cause psychosocial dysfunction that in turn contributes to distress, that the isolation inherent in psychosocial dysfunction might increase SP, or that distress might increase problematic SP that in turn causes psychosocial dysfunction. Anderson and Clarke's (2019) qualitative study supports these explanations and suggests that multiple explanations might be relevant, creating a cycle of SP, distress and psychosocial dysfunction.

### The current study

Attempts to understand the experience of problematic SP have offered preliminary insight into complex experiences such as consciousness, attention and shame. This research employs a qualitative approach to develop a fuller picture of the subjective experience of problematic SP, its processes and maintenance. It is hoped that this will inform nuanced intervention and help to contextualise and structure the work of psychological and therapeutic professionals.

## METHOD

### Participants

This research captures the experiences of 17 participants who self‐defined as being distressed by their problematic SP. The use of self‐definition reflects the ethos of valuing subjective meaning, concerns about the validity of diagnosis (Cosgrove & Krimsky, 2012; Pearce, 2014), and the hope to avoid priming or exclusion based on diagnostic assumptions. Subjective definition may also be representative of those self‐referring for psychological therapy.

Sixteen participants contributed a complete interview and two did not finish their interviews. One of those with an incomplete interview consented to the inclusion of their responses and has been included. This number of interviews was considered appropriate to ‘tell a rich story’ (Braun & Clarke, 2013) and represent a range of SP styles, such as ‘automatic’ and ‘focussed’ (Walther et al., 2009). Male participants were purposively recruited as researchers have suggested a gendered difference in the experience of problematic SP (e.g. Prochwicz et al., 2016), though ultimately only two males participated, perhaps reflecting apparent female predominance in problematic SP (e.g. Wilhelm et al., 1999). Participants were aged between 21 and 53 years (mean: 34 years; standard deviation: 10.2).

### Interviews

Semi‐structured interviews were chosen to offer a balance of structure and flexibility, providing space for unanticipated issues (Fylan, 2005), and accommodating variation in problematic SP presentations, while addressing the main areas of interest with all participants. Pre‐prepared questions covered the features and experience of problematic SP, coping, sense‐making and help seeking (see Box 1).

BOX 1 **Interview guide**

**Features of skin picking**

- To start with, I'd really like to know a bit about how you pick. Please could you describe to me how you typically pick your skin?
- Where on your body do you pick? Do you pick anywhere else on your body?
- What is it that you pick at, if anything? Do you have any diagnosed or undiagnosed skin conditions that impact your picking?
- How often do you pick?
- When do you tend to pick?
- How much or how long do you pick for?
- Does the picking hurt or damage your skin?
- Do you notice that you pick in different ways? What do you think changes/influences the different ways that you pick?
- Where are you when you pick (home, work etc.)?
- When did you start picking more than you'd like to?
- Has your picking changed over time?

**Experience of skin picking**

- How do you feel before/during/after picking?
- How do you feel about the fact that it happens?
- What do you feel about your skin in general?
- What kind of relationship do you feel that you have with your body? Does your skin impact how you see your body?
- How do you feel about any damage that you cause to your skin?
- What is it that you find distressing about picking?
- What difference would it make if you did not pick your skin?

**Coping with picking and distress**

- Do you do anything to manage your picking? Are there things that you have tried in the past?
- What helps you reduce/stop picking?
- Do you do anything to manage any skin damage?
- Do you do anything to help reduce the distress that you have relating to your picking?

**Sense making**

- Why do you think you pick?
- When is your picking particularly good/bad? Have you noticed any patterns?
- Do you do anything else that you consider similar to picking? In what ways are they similar?
- Have you come across the idea of skin picking as a disorder? How do you feel about it being described like this? Does it change the way that you see your picking?

**Talking about picking**

- Do you talk to other people about your picking?
- How is it to talk to me about it now?
- Why did you choose to be interviewed by (email, Skype, WhatsApp)?
- Do you think your answers would be different if I were interviewing you in a different way?
- Do you know anyone else who picks their skin? What do you think about their picking?
- Have you ever sought support?
  - YES – how was your experience? Was it helpful? What would you change?
  - NO – what stops you? What would be useful/unhelpful if you were to get support?

**Close:** Is there anything you'd like to say that we have not talked about?

As shame appears central in the experience of problematic SP for many (Anderson & Clarke, 2019) and is an inherently relational emotion (DeYoung, 2015), it seemed critical to consider how the interpersonal nature of the interview might impact upon participants' comfort and disclosure. As individuals manage shame in a variety of ways (e.g. perfectionism, withdrawal, gaining command of exposure of shameful experiences through humour; Kaufman, 1996), multiple interview modalities with varying degrees of anonymity were offered. Previous research suggests that the use of multiple modalities of interview may benefit recruitment and data quality (Heath et al., 2018) and be useful in exploring the experiences of hard‐to‐reach populations (e.g. Dures et al., 2011). Dures et al. (2011) used multiple interview modalities (face‐to‐face, telephone and email) in their study of the psychosocial impact of Epidermolysis Bullosa (EB), a rare skin disorder. The authors noted that given the stigma associated with visible difference in the wider culture, the fact that the participants' appearance could be seen by the interviewer (who did not have EB) when face‐to‐face may have impacted the content of these interviews (Dures et al., 2011). This may be relevant to the use of interviews when researching problematic SP.

Modalities offered in the current study were face‐to‐face (where practical), *Skype* (video call or audio only), telephone, email and instant messenger (IM). Face‐to‐face and *Skype* interviews are considered to benefit from visual cues and communication (Braun & Clarke, 2013; Hanna & Mwale, 2017), whereas email and IM interviews offer participants a greater sense of felt anonymity and reduce the chance of visual scrutiny by the researcher (Braun et al., 2021), and may be useful where participants find face‐to‐face interviews difficult (Deakin & Wakefield, 2014). Different modalities offer varying degrees of synchronicity, and asynchronous methods may be preferred by those wanting to moderate their disclosure (Gibson, 2017). In the current study, most participants opted for IM interviews using either *WhatsApp* or *Facebook Messenger* (combined *N* = 12), two chose email, two telephone; only one participant requested a face‐to‐face interview. As face‐to‐face was not practical in this instance, this participant was interviewed via *Skype* video call. No incentives were offered for participation.

### Data collection

Information on the study was provided on a website to allow participants to access the information anonymously and without obligation. The website was promoted on two international SP support groups on *Facebook*, and later on *Twitter*, requesting UK‐based participants. The *Qualtrics* online survey platform (https://www.qualtrics.com) was used for participant screening, consent and recording interview modality preference. Interview lengths varied from 90 min for the shortest telephone interview to 3 months for the longest asynchronous IM interview. The interviews were conducted by the first author who was a trainee counselling psychologist with experience of researching problematic SP (Anderson & Clarke, 2019).

### Data analysis

Reflexive thematic analysis (TA) was used to develop patterns of experience and themes from the participants' standpoint (Braun & Clarke, 2006, 2013). The analysis was underpinned by a critical‐realist ontology, assuming the existence of a meaningful reality while acknowledging the impact of factors such as participants' culture and language on the experience and expression of this (Ussher, 1999). *Skype* and telephone interviews were orthographically transcribed. Email and IM interviews, already in textual form, were copied verbatim into a datafile. These data were not edited other than correcting self‐evident typos for ease of reading and participants were allocated pseudonyms, which are used below when quoting from individual interviews.

As data collection took place over a protracted length of time, coding began before the full dataset was available. Coding and analysis were led by the first author and guided by Braun and Clarke's (2006, 2013) approach to TA. Initially, transcripts were read and re‐read, and notes made. Transcripts were then read closely and initial coding labels were written alongside in the margins. Codes were refined and added to as interviews were revisited over time. The second author coded three interviews, and the first and second authors discussed and reflected on differences in coding, and this discussion informed the first author's ongoing coding of the data. Once coding was complete, the first author developed larger patterns across the dataset and grouped the codes into potential themes (Braun & Clarke, 2006), striving to allow a relatively inductive approach unsteered by previous research and definitions.

The first author made a ‘directory’ of themes accompanied by the related participant quotations, making it possible to review themes and consider their ‘fit’ with the data. Writing was an integral part of the analysis (Braun & Clarke, 2013), helping to finalise theme structure and boundaries. Table 1 provides an overview of the thematic structure.

**TABLE 1:** Thematic structure

Theme title	Summary	Sub‐themes
The Voice that Permits Skin Picking: Cognitions Drive Skin Picking and Undermine Resistance	Participants' SP behaviour was driven by cognitions and circumstances that permit or accommodate SP, and that diminish the will to stop.	Skin Texture Must Go ‘Oh Well’ and ‘So What’: Resignation to Skin Picking Skin Picking Because I Can: Permissive Circumstances
Switching Everything Else Off	Participants described their SP as having dissociative qualities. SP often occurred alongside feelings of stress or distress, and the experience of ‘zoning out’ offered by SP gave participants relief.	Zoning In to Zone Out It Comes with Negative Emotions Reducing Emotional and Mental Noise
I Worry About People Looking and Judging Me: Distress in How Skin Picking is Seen	Much of participants' distress seemed mediated by beliefs about the appearance of SP and its damage in the eyes of others. Participants were self‐conscious of SP damage and felt that their SP was misunderstood by others.	Shame of Skin Picking I am Misunderstood

## ANALYSIS

### The voice that permits skin picking: cognitions drive skin picking and undermine resistance

Throughout the interviews, participants described conflict between the drive to skin pick and to stop, sometimes describing it as a ‘running argument’ (Marcus) or ‘battle’ (Olivia) between separate voices or ‘minds’ (Melanie). This theme describes how participants rationalised, justified and permitted their SP in the moment, despite the distress that often followed.

#### Skin texture must go

Cognitive processes, such as thoughts and attention focussed on the skin, seemed to both trigger SP and make it difficult to stop once started. Participants unanimously described the want to smooth skin texture as an immediate precipitator of SP, and saw resulting skin damage as unintentional, collateral harm. Julie described how her desire to smooth texture meant that her skin could not heal:

When the skin that's been picked goes hard, I'm compelled to pull at that too, because I hate the feel of lumps or unevenness on my fingers. This means the worst wounds are pretty much never healed.

While a few participants considered there was ‘no thought process to [SP]’ (Rebecca), others considered texture to be ‘not acceptable’ (Melanie) or ‘gross’ (Sam), and some implied that texture was unhygienic by using words such as ‘cleaning’ (Annie, Lisa) to describe SP. Some described how scrutinising social experiences made them ‘more alert for imperfections’ (Ellen), and some suggested that others had influenced their view of skin texture, such as through bullying or encouragement to skin pick. Participants were able to view these thoughts about skin texture with critical distance when not engaged with SP, acknowledging that they did not ‘reflect reality’ (Olivia) and that SP achieved ‘the opposite’ (Eden) of their intentions.

Many described SP to involve a ‘[detailed] level of focus’ (Ellen) that maximised sensory information about the skin and caused them to ‘see something else’ (Olivia), perpetuating SP. Several participants described attempts to moderate SP by reducing this sensory information (as with ‘stimulus control’ techniques; e.g. Jafferany & Patel, 2019), though participants' motivation to skin pick often overcame these barriers as they ‘cheated’ (Lucy) or ‘relented’ (Rebecca).

Some participants described struggling to stop an episode of picking as they felt drawn to having skin picked to a finite point, such as ‘when something comes out’ (Leanne) or ‘all uneven surfaces are gone’ (Sky). It was not always clear whether these cognitions were separate from the general motivation to smooth skin (someone set against texture may want to remove all rather than some), but some seemed motivated specifically by a sense of completion. Sky explained that ‘I'll end up carrying on because it's like I've not finished’, and Lisa described satisfaction in feeling ‘really diligent’ when picking her skin completely and ‘perfectly’. The appeal of completion logically seems related to the idea that incompleteness may be uncomfortable for people with problematic SP (Snorrason, 2016).

#### ‘Oh well’ and ‘So what’: resignation to skin picking

Several participants described cognitions permitting SP that were characterised by resignation or a lack of resistance to it. Rebecca explained that she would ‘have to pick it sooner or later, so I might as well get it over and done with’, suggesting that she considered SP to be inevitable and resistance to be futile. Others diminished the significance or impact of damage from SP, such as where skin was already disliked or damaged from previous SP. Jenny related the start of her SP to dislike for her body, recalling ‘so what to it if it was going to make my legs look a mess, because nothing would have made me like my body so why not do something that could cause scars or whatever’. Jeff similarly connected feelings of low self‐worth to a resigned acceptance of SP:

I'm feeling down at the moment, being hard on myself so I start picking… I think I take out my frustration about feeling low by picking or biting myself. It's almost like I don't matter so if I hurt myself that's fine.

A few participants picked more or more aggressively at hidden areas of the body where ‘no one will ever see [the damage]’ (Lisa), suggesting that concealable damage was more tolerable. This echoes Rehm et al.’s (2015) qualitative study of hair pulling which found that permission‐giving thoughts were common, and one participant described how the ability to conceal bald areas ‘triggers off the urge’ (p. 221). A couple of participants in the current study described purposefully planning activities that exposed their skin to others as an incentive to stop SP.

Some described SP as being more acceptable and less resisted where it was more manageable, such as where the impact on everyday life was tolerable or may be mitigated, or where it was felt to serve a beneficial or ‘adaptive’ (Lucy) function, such as providing emotional relief. The impact of manageability was particularly apparent in how SP often *wasn't* allowed where it was *un*manageable. For example, Marcus talked about how he ‘steers clear of my legs now’ due to infection and ulceration, implying that this damage was a deterrent, whereas more moderate damage was manageable and harder to resist.

#### Skin picking because I can: permissive circumstances

Skin picking was often described as a ‘default’ behaviour, gravitated towards where it could be accommodated or where measures were not actively taken to resist it. Leanne considered that ‘if I'm in a situation where I can, then I do’, suggesting that being ‘able’ to skin pick was a direct predictor for SP, and Sam explained that ‘if im not distracted by something else then i am [picking] or thinking about picking’. Several participants described SP more when bored, where it offered ‘something for my hands to do’ (Rebecca). Participants similarly described SP less when busy, either due to distractions or having fewer opportunities to skin pick, and a couple of participants deliberately limited their free time to reduce opportunities for SP. Previous research into ‘habit disorders’ notes that habits increase in times of passivity or waiting, speculating that this relates to unoccupied hands (O'Connor et al., 2003).

Two alternative explanations were given for the relationship between boredom and SP. Firstly, Aisha felt that SP offered relief from boredom as a positively uncomfortable experience, by similar means that SP relieved stress (see below). Secondly, Olivia associated boredom with being alone, feeling that a lack of social company allowed her to become more ‘self‐focussed’, increasing her SP. Both typical and problematic SP are reported to occur largely in private (Bohne et al., 2002) and in situations of reduced socialisation (O'Connor et al., 2003), and visibility to others seems to inhibit SP (see previous subtheme). Additionally, social connection may mediate emotional regulation (e.g. Seppala et al., 2013), and isolation may increase self‐defeating and addictive behaviours (Hari, 2015; Twenge & Baumeister, 2005). Although research has identified boredom as a precipitant of SP (e.g. Bohne et al., 2002), to our knowledge the mechanisms of this relationship have not been explored.

### Switching everything else off

This theme describes how participants reported SP to have dissociative qualities that offered emotional and mental relief.

#### Zoning in to zone out

Over half of the participants described intense attention on the process of SP, using words such as ‘entranced’ (Annie) and ‘pure focus’ (Marcus) to describe how their attention was consumed. Melanie explained that ‘in that moment in time nothing else matters apart from what your picking and where your picking’. This idea of focus seems to relate to Wilhelm et al.’s (1999) use of the word ‘mesmerised’ and Deckersbach et al.'s (2003) description in their case study of ‘zooming in’ (p. 255) in front of the mirror, and seems implicit in a ‘focussed’ style of SP (Walther et al., 2009). The current participants' descriptions also seem evocative of Heatherton and Baumeister's (1991) description of ‘cognitive narrowing’ during binge eating, whereby individuals narrow ‘the focus of [their] attention to the present and immediate stimulus environment’ (p. 88).

Where attention is focussed on SP, it correspondingly disconnected from other thoughts and senses. This zoning out or ‘trance state’ (Melanie) has been briefly mentioned in research relevant to problematic SP (e.g. Wilhelm et al., 1999). In the current study, zoning out was mentioned by almost all participants. Lisa explained:

I feel like I'm just switching everything else off… it's all about the picking when I'm picking, it's not about anything else… it's blocking everything out for a minute. It's like putting the pause button on almost, stopping the world.

Julie described how ‘there can be trance‐like times when I lose track of time picking at my head’ and Annie *described* ‘rarely [feeling] any sort of pain’. This disconnect was sometimes implicit in participants' descriptions of suddenly realising the extent of SP.

Participants' disconnect was sometimes indicative of depersonalisation. Ellen forgot that ‘what I'm doing is attached to my actual face’ and Lisa said that her skin ‘may as well be something lying next to me’, seemingly considering their skin as object rather than part of a living subject. This corresponds with theories in body dysmorphia that suggest attention to be consumed by the body's object image at the expense of bodily senses (Veale, 2004). It may be that disconnect from awareness time, pain and subjectivity reduces access to motivation to stop SP, as has been similarly suggested in binge eating (Heatherton & Baumeister, 1991).

The extent of disconnect varied across participants. Some described access to thoughts about stopping SP, creating an internal argument or ‘battle’ (Olivia) between separate voices or different ‘bit[s] of my brain’ (Leanne). Melanie gave words to her two perspectives:

I would say I have two minds when I'm in that trance like state [picking], it's like I have an angel and a devil, the angel is telling me to stop picking I'm only making it worse and that it's hurting me I have to stop and then there's the devil telling me to keep going look how much you've picked and how much stuff you've got out your skin it makes me feel better, I'm usually bleeding a lot before I'll convince myself that it's time to stop and step away…

Thoughts about stopping were often outcompeted by the drive to pick and did not translate into action: ‘During [picking], I quite often realise I'm doing it and will tell myself to stop but won't physically be able to snap myself out of it’ (Eden).

Discussion of attention is complicated by subtleties, changes and contradictions within participants' descriptions, captured in Ellen's portrayal of being ‘entirely absorbed on one level, klaxons going off on another’. Participants' consciousness, thoughts and awareness during SP were clearly not simple nor singular experiences.

#### It comes with negative emotions

Uncomfortable emotions, most often those carrying a nervous energy such as stress or anxiety, were described by all but one participant as increasing SP frequency and/or aggression. Many considered or suspected SP to be in part a response to deeper or broader emotional issues, with several connecting SP to traumatic experiences such as bullying, loss and abuse. Lucy considered her worst SP to have been ‘a symptom of the distress, despair and utter hopelessness’ that she felt when subject to an abusive relationship. Similarly, Leanne suspected that her SP was ‘a symptom of that anxiety, the restlessness, the feeling of never being truly peaceful’ following a traumatic loss. Marcus described how the stress of SP damage itself precipitated further cycles of SP: ‘[picking damage makes me] feel a burden, waste of space, and it perpetuates the cycle all over again’. This impact of emotion on SP corresponds with the findings of previous research (e.g. Neziroglu et al., 2008; Roberts et al., 2015) and the notion that stress increases risky and instinctively driven decision making (Starcke & Brand, 2012). Some participants suggested that positive systemic change (such as changes in relationships) or stress management helped to reduce SP, though SP often persisted, having become a problem ‘in its own right’ (Marcus) and leaving participants with ‘more of a habit’ (Helen).

#### Reducing emotional and mental noise

Participants connected emotion and SP in several ways, such as that stress caused more self‐touching, which meant more texture was noticed, or that mood impacted how imperfections were appraised. The most common connection was that the trance state of SP served an emotional function by giving respite from thoughts and feelings. Participants described SP as a ‘reliever’ (Sky) and as ‘therapeutic’ (Jenny). Lucy, who considered her SP to be a stimming behaviour (patterns of repetitive, purposeless movements [Mackenzie, 2018], which are felt to regulate sensory overload and uncontainable emotion [Kapp et al., 2019]) associated with autism, explained how her SP reduced overwhelm:

When I'm in an environment with bright lights and lots of people talking (and this gets amplified greatly by stress and/or tiredness) – everything is just too much and I guess the stimming (which the biting/picking [at skin] is part of) redirects my attention onto something I can handle when I can't handle the world around me.

This perspective echoes the stimulus regulation model of hair pulling, where hair pulling is considered an attempt to balance senses and stress (Penzel, 2003). Similar theories have been proposed for autistic people, where repetitive behaviours may regulate sensory overload and uncontainable emotion (Kapp et al., 2019). Marcus vividly described the calming effect of SP:

My thought process [when overwhelmed] becomes like a carousel at the fair, blurred images whizzing past my eyes unable to pick a thought and hold it down long enough to process it. Then in the madness the only thing that quietens the mayhem is picking. The act of picking focuses my mind in a singular thought and task, giving my brain a much needed break from itself…

This zoning out helped participants to forget about ‘whatever else is going on’ (Jenny) and ‘take a break from life’ (Sky), using similar language to a participant in Rehm et al.’s (2015) study who described hair pulling as ‘a break from thinking’ (p. 219). Lisa suggested that SP allowed her to better process her thoughts and let ‘the experiences of the day just sort of filter through’. Around a third of participants described SP as a coping behaviour, such as that it ‘helped me out of what I needed to be out of at the time’ (Aisha). Several considered SP to be preferable to ‘alternatives' such as self‐injury and smoking.

### I worry about people looking and judging me: distress in how skin picking is seen

While the direct consequences of SP (such as pain and loss of time) were problematic and distressing, distress relating to beliefs about the appearance of SP in the eyes of others was powerful across the interviews.

#### Shame of skin picking

Shame and self‐consciousness about the appearance of SP damage and/or behaviour were present to varying degrees across all interviews. For some, the appearance of damage was their primary, if not only, concern: Ellen remarked that ‘if it didn't leave a mark I genuinely wouldn't care in the slightest’. Concerns about others' thoughts or judgement were sometimes more implicit, such as in efforts to conceal damage or avoid exposing activities. Helen described not answering the door after ‘a bad pick’, explaining that ‘if I do have to go out my anxiety is through the roof… [I worry about] people looking and judging me… wondering what is wrong with me’. For some, shame focussed on the aesthetic appearance of SP damage, which made them feel or look unattractive. Others feared that skin damage would be mistaken for disease or something contagious, corresponding with thinking about stigma relating to pathogen avoidance (e.g. Kurzban & Leary, 2001) and echoing the experience of people living with skin conditions (Hrehorów et al., 2012; Kellett & Gilbert, 2001).

For some, the problem with the damage was that it revealed the act of SP, which all participants wanted to hide to some extent and Jenny called a ‘dirty little secret’. Very few participants articulated specific cognitions relating to their shame. Those that did suggested SP to be childish, reflect poorly on their self‐care or represent a lack of self‐control. This latter point was evident in how several participants described making ‘excuses’ (Rebecca) for damage, suggesting that accidental damage would be less shameful. Leanne made this point explicitly:

It's not something that has happened to me by accident that was out of my control, I wouldn't be ashamed then, so it's got to be that it's something I've physically done to myself and I'm ashamed that I haven't been in control of myself enough to not do it.

#### I am misunderstood

Many participants expressed frustration that others advised them to ‘just stop’ SP, feeling that their SP was misunderstood as being easy to stop. Jeff found these comments shaming as it was ‘like it's something I should be able to control and is easy for me to stop doing’, suggesting that SP was seen by others to reflect a weak will. Bradley and Ecks' (2018) study of family relationships alongside hair pulling similarly described parents commenting ‘why can't you just stop’? (p. 572), serving to only increase family tensions and hair pulling. In the current study, several participants also expressed frustration that SP was considered a ‘habit’, a word seeming to diminish the complexity and tenacity of SP.

Feeling misunderstood was often silencing. Participants often felt that talking about SP risked negative judgement or that a lack of understanding made conversations ‘more hassle than [they're] worth’ (Rebecca). Many were reluctant to seek professional support and past help‐seeking experiences were mixed; some told positive stories of professionals recognising their distress and working to understand them, while others, such as Jenny, described feeling dismissed:

I saw a psychiatrist and when I mentioned it to him and showed my legs to him he pretty just ignored what I said and started talking about something else! … it was annoying because to me it is a problem, yet he just made it seem like it was just nothing… I'd just like them to take it seriously, rather than making me feel like I'm just being silly and it's not a problem at all.

It was common for participants to feel that helping professionals should have a pre‐existing understanding of SP, considering that this would aid treatment, help them ‘to be understood’ (Olivia) and mean that SP would be treated ‘as a serious issue’ (Leanne). This demonstrates the power of the clinician and how a response lacking in understanding may be shaming (Lazare, 1987).

## DISCUSSION

The current study explores three themes in the problematic experience of SP, each suggesting opportunities for therapeutic intervention and support.

‘The Voice that Permits Skin Picking’ considers the cognitions that many participants identified as driving their SP or weakening their resistance, as well as the environmental factors (e.g. being alone, sedentary activity) that permitted SP. It seems imperative that therapists are prepared to support individuals to understand and manage this immediate drive to pick. This drive to pick has been the central target for previously suggested therapies such as Habit Reversal Therapy, Cognitive Behavioural Therapy (CBT) and Acceptance and Commitment Therapy (ACT) (e.g. Schuck et al., 2011; Twohig et al., 2006). These therapies aim to recondition, restructure or loosen the impetus to pick, though discussion of the cognitions and behaviours that may be targeted is limited (e.g. Deckersbach et al., 2002). Findings from the current study suggest patterns in participants' cognitions, such as that (paraphrased) ‘texture is unacceptable’, ‘I need to get it all’, ‘it's going to happen eventually’ and that ‘the damage doesn't matter’ (either ‘it won't be seen’ or ‘I didn't like my body anyway’). These thoughts often seemed influenced by factors such as social experiences, attention, sensory information, how much the body or self was valued, self‐focus, inoccupation or the ability to conceal damage. An understanding of these precipitants may be particularly helpful for therapists developing interventions seeking to reduce SP. For example, having time and space to pick may be targeted by behavioural interventions intending to fill time positively or engage socially at triggering times. ‘I didn't like my body anyway’ might warrant an exploration of their relationship with their body, and benefit from interventions to grow self‐compassion and esteem. Because of the ability to hide damage, SP may be sensitively challenged by goals to stop hiding, as used by Deckersbach et al. (2002) in an individual's treatment.

‘Switching Everything Else Off’ explores how the attentional experience of SP offered escape from emotion and overwhelm. This locates possible problems in contextual emotion and overwhelm (suggesting that contextual emotion or systemic factors may be considered in therapy), and/or in the use of SP as a problematic coping mechanism (suggesting that it may be helpful for therapy to strengthen alternative coping strategies). Further, this suggests that failure to address emotion or develop healthy coping risks SP being replaced with alternative unhealthy strategies.

While problematic SP has been previously suggested to have an emotional function (Flessner & Woods, 2006), the mechanisms of relief have not to our knowledge been explored. Participants in the current study give rich descriptions of this experience, suggesting that focus on the repetitive and satisfying task of SP may facilitate zoning out away from competing thoughts and stress. An understanding of these processes may be helpful for therapists when considering which alternative coping strategies might offer similar relief (such as those involving repetitive, rewarding behaviour). The analysis presents similarities in descriptions of self‐regulation in SP and autistic stimming (see Kapp et al., 2019), though this observation is preliminary and requires further exploration.

‘I Worry About People Looking and Judging Me’ highlights shame and the real or imagined perspectives of others as significant in SP distress. When considered alongside the two other themes, this distress may drive further SP by increasing self‐criticism and thus strengthening permission giving cognitions, as well as increasing the allure of a zoned‐out headspace. As well as impacting on quality of life, social avoidance relating to shame may also increase factors such as aloneness or boredom that may precipitate or accommodate SP.

Despite the apparent centrality of distress in the problematic experience of SP, treatments have largely targeted the *urge* to pick (e.g. Flessner et al., 2008; Schuck et al., 2011). It seems imperative that therapists might also consider distress as itself a target of therapy; a reduction in distress, as well as itself being positive, may also weaken the mechanisms and cycles that drive or exacerbate SP. Distress in the current study related significantly to appearance. Clarke et al. (2014) recommend applying CBT principles to appearance‐related distress and the cycles that maintain it, and ACT may also be useful for working with issues relating to visible difference (e.g. Shepherd et al., 2019; Zucchelli et al., 2018).

The centrality of shame in SP distress, and its interpersonal nature implicate the role of the therapist in ameliorating or worsening distress. The current study shows how dismissive professionals may be shaming and silencing, suggesting that empathy for and acceptance of SP behaviours and distress may be powerful therapeutic tools. The current study reports self‐objectification, depersonalisation and aesthetic self‐criticism in the experience of SP. This underlines the importance of the therapist not objectifying the client, such as by viewing the skin solely as an object to be fixed, and the imperative of seeing and welcoming the client's whole subjectivity, including the side of them that is drawn to SP. It may also be helpful to build social support within understanding communities, such as the online groups that many of the current study's participants praised, or group psychotherapy as recommended by Nakell (2015).

## CONCLUSION

This study offers insight into the mechanisms of the appeal, tenacity and distress associated with SP. It presents SP not simply as a problem of drive and behaviour, but one of emotional context and consequence. The complexity of participants' experiences suggests that cases may be best understood by considering and responding to what is meaningful to the individual. The transdiagnostic fields of appearance, shame and body shame, impulsivity and emotion regulation may flexibly inform this work. Non‐judgemental therapy that accepts all elements of the individual, including those which the individual rejects, may provide the space needed to develop insight into SP.

## AUTHOR CONTRIBUTIONS

**Suzy Anderson:** Conceptualization; data curation; formal analysis; investigation; methodology; project administration; writing – original draft; writing – review and editing. **Victoria Clarke:** Conceptualization; formal analysis; methodology; supervision; validation; writing – review and editing. **Zoe Thomas:** Conceptualization; supervision; writing – review and editing.

## CONFLICT OF INTEREST

All authors declare no conflict of interest.

## Data Availability

The authors elect to not share data.
